# SGDO-SLAM: A Semantic RGB-D SLAM System with Coarse-to-Fine Dynamic Rejection and Static Weighted Optimization

**DOI:** 10.3390/s25123734

**Published:** 2025-06-14

**Authors:** Qiming Hu, Shuwen Wang, Nanxing Chen, Wei Li, Jiayu Yuan, Enhui Zheng, Guirong Wang, Weimin Chen

**Affiliations:** School of Mechanical and Electrical Engineering, China Jiliang University, Hangzhou 310018, China

**Keywords:** simultaneous localization and mapping (SLAM), dynamic environments, depth consistency constraints, static quality weights, vision sensor

## Abstract

Vision sensor-based simultaneous localization and mapping (SLAM) systems are essential for mobile robots to locate and generate spatial models of their surroundings. However, the majority of visual SLAM systems assume static settings, leading to significant accuracy degradation in dynamic scenes. We present SGDO-SLAM, a real-time RGB-D semantic-aware SLAM framework, building upon ORB-SLAM2 to address non-static environments. Firstly, a multi-constraint dynamic rejection method from coarse to fine is proposed. The method starts with coarse rejection by combining semantic and geometric information, followed by detailed rejection using depth information, where static quality weights are quantified based on depth consistency constraints. The method achieves accurate dynamic scene perceptions and improves the accuracy of the system’s positioning. Then, a position optimization method driven by static quality weights is proposed, which prioritizes high-quality static features to enhance pose estimation. Finally, a visualized dense point cloud map is established. We performed experimental evaluations on the TUM RGB-D dataset and the Bonn dataset. The experimental results demonstrate that SGDO-SLAM reduces the absolute trajectory error performance metrics by 95% compared to the ORB-SLAM2 algorithm, while maintaining real-time efficiency and achieving state-of-the-art accuracy in dynamic scenarios.

## 1. Introduction

Simultaneous localization and mapping serves as a foundational technology in robotics. This technique can help robots in unknown environments to localize their position and perform positional environment construction [1]. It has been widely used in the fields of autonomous driving, augmented reality (AR), virtual reality (VR), and drones [2]. The commonly used sensors for SLAM are LiDAR sensors and vision sensors [3]. Vision sensors are lighter, lower cost, and have rich semantic information [4]. As a result, visual SLAM turns out to be a research hotspot [5]. Visual SLAM systems use the data obtained from the visual sensors for simultaneous estimation of the motion state as well as for map construction and environment model updating [6]. Classical visual SLAM algorithms such as ORB-SLAM2 [7], VINS-Mono [8], ORB-SLAM3 [9], and LSD-SLAM [10] enable accurate pose estimation and map construction in static settings. However, non-static elements like moving pedestrians and vehicles in real-world settings can disrupt data association accuracy, cause significant localization errors, and even lead to localization failure [11].

Therefore, reducing interference from dynamic objects has become an important research topic in visual SLAM. Researchers initially used geometric information to minimize dynamic object interference. For example, random sampling consistency (RANSAC) [12] iteratively fits a static scene model by rejecting dynamic objects as anomalies and retaining inliers. Epipolar geometric constraints are also used to reject outliers. This method calculates how much matched points deviate from these constraints, discarding those that violate the base matrix’s geometric conditions. However, relying solely on geometric information for dynamic rejection forces the system to perform iterative computations. This process is computationally expensive and inefficient, and it struggles to guarantee real-time capability in SLAM systems. Recent breakthroughs in deep learning have motivated efforts to fuse semantic understanding with VSLAM frameworks [13]. Techniques like object detection [14,15] and semantic segmentation [16,17] enable the acquisition of scene semantics for enhanced environmental understanding. Purely semantic-based dynamic rejection methods risk leaving residual dynamic interference in real-world environments. This occurs because they cannot detect new dynamic object types outside their training data. Furthermore, in complex scenes with semantic coupling between dynamic and static regions, over-reliance on semantic segmentation may erroneously remove static points containing valid geometric features. This leads to accumulated pose estimation errors, degrading SLAM robustness and map completeness. Therefore, some recent studies combine geometric and semantic information methods to suppress dynamic interference, such as [18,19,20,21,22]. Some other studies take semantic information as prior information and construct dynamic and static probability models to select static features for pose estimation, such as [23,24]. These algorithms effectively address traditional single-modal limitations in dynamic feature discrimination. Through synergistic optimization, they enhance dynamic perception robustness and maintain real-time performance via a lightweight fusion architecture, improving positioning accuracy. However, these algorithms exhibit limited dynamic perception in specific scenarios. For instance, objects moving along the polar direction (uniformly moving unlabeled targets) may have motion compensation residuals that mimic static features, which the semantic model fails to recognize due to missing category labels, reducing system accuracy. Additionally, these methods lack a refined quality assessment of retained static features. Features with latent motion trends or discontinuous observations may persist, further compromising reliability.

This paper presents a semantic visual SLAM system based on rejecting dynamic features from coarse to fine and static weight optimization. A multi-constraint dynamic feature point rejection method is highlighted. Firstly, the semantic labels and geometric consistency are combined to coarse cull. Fine culling is then performed using deep consistency constraints, and static quality weights are quantified. This method improves dynamic perceptibility and can reject dynamic features more accurately, which enhances the positioning stability of the system in complex environments. This paper also investigates a back-end static point optimization method driven by static quality weights. The method increases the weights of high-quality static features for pose optimization, which indirectly improves the trajectory estimation reliability of the system. Sparse maps and dense point cloud maps are also constructed to adapt to different environmental requirements. SGDO-SLAM improves the absolute trajectory error performance metrics by 95% in highly dynamic sequences of the TUM dataset as compared to ORB-SLAM2.

The main contributions of this paper include the following:

(1) We propose a semantic visual SLAM system base, called SGDO-SLAM, based on multi-constraint rejection and static quality-weighted optimization. Compared to existing advanced methods, SGDO-SLAM has higher accuracy in dynamic environments. The system also builds semantic point cloud maps to assist the robot in understanding the environment.
(2) A dynamic feature rejection strategy from coarse to fine is proposed. Coarse culling is first performed by combining priori semantic information with the results of the epipolar geometric constraints. Then, finer rejection is performed by projecting the feature points in the current frames to the key frames according to the depth consistency constraints. Meanwhile, static quality weights are quantified and used to assess the merits of static features.
(3) A static weighting-based optimization method for the SLAM back end is proposed. The method is based on the front-end static quantization weighting drive, which distinguishes the static superior and inferior features after the dynamic features are rejected. High-quality visual features are prioritized for pose estimation and back-end optimization. This approach ensures accurate localization and high-quality mapping.

The remainder of the paper is organized as follows. Section 2 describes the work related to the system. Section 3 describes the specific ways in which the system is implemented. Section 4 provides an experimental evaluation and analysis of the results. Section 5 summarizes the core insights of this work and highlights emerging questions for future exploration.

## 2. Related Works

Dynamic object rejection has emerged as a critical challenge in visual SLAM research. Current methodologies for identifying and addressing these environmental perturbations can be grouped into three primary approaches: methods based on geometric information, methods based on semantic information, and fusion methods.

### 2.1. Methods Based on Geometric Information

Geometry-based methods typically eliminate dynamic features through rigid geometric constraints or motion conformance check. Kundu et al. [25] established epipolar geometric constraints by constructing fundamental matrices, classifying features as dynamic when their epipolar distance exceeds empirical thresholds. Yang et al. [26] developed a modified RANSAC-based approach that coarsely removes apparent dynamic points, computes the fundamental matrix with remaining points, and then performs secondary elimination using epipolar constraints. Zou and Tan [27] employed triangulation and feature tracking reprojection error analysis to discard features with excessive residuals. Studies [28,29] directly detected motion patterns through optical flow fields and residual analysis. While geometric constraint-based dynamic SLAM methods retain basic localization in specific dynamic environments, they rely on computationally intensive feature matching and iterative optimization. Under complex motion patterns, occlusions, or illumination changes, their complexity sharply rises, degrading both real-time performance and positioning accuracy.

### 2.2. Methods Based on Semantic Information

Semantic-based approaches typically employ convolutional neural networks (CNNs) to identify potentially dynamic objects in images, followed by selective removal of feature points within detected dynamic zones. Zhang et al. [30] implemented YOLO-based object detection to identify dynamic entities, subsequently eliminating associated feature points through semantic-guided filtering. Sheng et al. [31] leveraged Mask R-CNN for semantic segmentation, classifying pixels as static features only when none of their four-connected neighbors exhibited dynamic characteristics. Xiao et al. [32] developed Dynamic SLAM, which discards all features within detected bounding boxes to mitigate dynamic object interference. Liu et al. [33] introduced RDS-SLAM, incorporating a dedicated detection thread with motion probability updates for semantic refinement, thereby enhancing computational efficiency. Lu et al. [34] optimized operational efficiency by fusing the semantic annotation of YOLOv10n with ORB-SLAM2 feature extraction, and dynamically rejecting moving object feature points to construct static maps. These deep learning-enhanced methods demonstrate improved localization accuracy through the effective identification and removal of most potentially dynamic objects. However, the performance of purely semantic dynamic rejection methods is limited by the predefined category coverage and scene semantic coupling strength. On one hand, for unknown dynamic targets outside the trained set, given the lack of a priori semantic knowledge support, the system is unable to build an effective dynamic model, resulting in constant residual unknown interference in the open environment. On the other hand, in the region of the dynamic–static boundary, the synergistic effect of semantic segmentation confidence thresholds and missing geometric constraints may incorrectly reject stable static feature points.

### 2.3. Fusion Methods

To address the inherent limitations of standalone semantic or geometric approaches, recent studies have introduced hybrid frameworks that synergistically combine both modalities to enhance robustness and efficiency. Bescos et al. [18] fused Mask R-CNN-driven instance segmentation with cross-view geometric constraints to address dynamic content. Pan et al. [35] employed Mask RNN coupled with an optimized polar coordinate geometric model for image segmentation, followed by kernel principal component analysis (KPCA) for point cloud denoising and octree-based dynamic filtering for refined data processing. He et al. [36] proposed DIO-SLAM, which utilizes YOLACT for scene target differentiation, integrates optical flow residuals to characterize moving objects, dynamically classifies rigid-body masks via optical flow consistency, and mitigates detection errors through motion frame propagation. Wu et al. [20] combined YOLOv3-based dynamic object detection with depth-enhanced RANSAC for dynamic feature removal. Jiao et al. [37] computed feature motion probabilities using inter-frame displacement analysis and removed high-probability dynamic features. Chang et al. [21] implemented a two-stage approach using YOLACT-based segmentation for preliminary dynamic filtering, supplemented by optical flow compensation for missed detections. While these methods demonstrate efficacy in general dynamic environments, they exhibit inherent limitations in complex motion scenarios. For example, non-potentially moving objects moving along the poles will lead to a decrease in the dynamic sensing ability, reducing the positioning stability. These methods do not provide a fine-grained quality assessment of the static features after dynamic reject processing. Pseudo-static features (such as slow-starting objects) and low-confidence static features may be present in the static feature collection. Uniform weighting of static features during subsequent processing can propagate errors, compromising overall system precision.

To address these issues, we propose a dynamic rejection strategy based on multiple constraints. After combining semantic labels and geometric constraints for coarse rejection, depth consistency constraints are used for more detailed rejection. This method improves the dynamic perception ability of the system and enhances the accuracy of rejecting dynamic features. Moreover, we also propose a back-end optimization method based on the static quality weights driven. It can allocate various weights for optimization based on the quality of the static points and reduce the influence of pseudo-static features and low-confidence static features on the optimization process.

## 3. System Overview

This section describes the specific implementation of the SGDO-SLAM system from six aspects. First, the general framework of the system and the basic implementation process are introduced. Second, the added YOLOv8 instance segmentation thread is briefly introduced. Then, the computational principles of the epipolar geometric constraints are presented. Next, the depth consistency constraint fine rejection dynamic feature method is introduced. Fifth, quantification of static quality weights and back-end static point optimization methods are presented. Finally, the creation of semantic point cloud maps is introduced.

### 3.1. System Architecture

**Figure 1:**
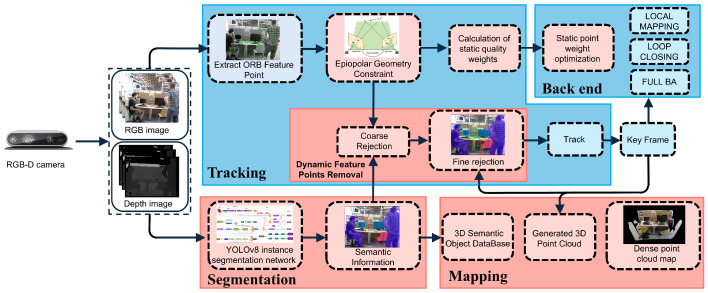
Overall architecture diagram of SGDO-SLAM system. The blue background shows the frame of the baseline algorithm ORB-SLAM2. The red background shows our main improvement work.

The framework diagram of the SGDO-SLAM system is shown in Figure 1. SGDO-SLAM extends ORB-SLAM2 [7] by augmenting its core architecture, tracking, local mapping, and loop closure threads, with two parallel modules: an instance segmentation thread and a semantic point cloud mapping thread. The multi-threading mechanism has been shown to enhance the efficiency of system operation. The framework employs an RGB-D sensor to capture synchronized color and depth streams in dynamic scenes, enabling real-time perception of non-static environments. Captured visual data are distributed in parallel to the tracking thread and the semantic segmentation thread. In the initial processing stage, the tracking thread detects feature points from the input frames, establishing foundational correspondences for subsequent pose estimation. Coarse dynamic feature rejection is then performed based on the semantic a priori information obtained from the segmentation threads combined with epipolar geometric constraints. After that, the remaining feature points in the processed image frames are projected into the corresponding keyframes, the second detailed rejection is performed according to the depth consistency constraint rejection strategy, and the static weight values of the remaining static points are output. The static weight values are passed to the back-end for the robust optimization of static points, and the constraints of good-quality feature points are added to optimize the position. In the semantic point cloud construction thread, the dynamic points in the depth map are removed by the result of instance segmentation, and semantic information is added to the point cloud map to enable more accurate localization and navigation in dynamic environments.

### 3.2. Instance Segmentation

Advancements in convolutional neural networks have driven significant improvements in detection and segmentation methods, achieving enhanced accuracy and computational efficiency. The task of the object detection algorithm is relatively simple, classifying the objects in the image and giving the object location and the detection box, which allows the use of a more lightweight network. Traditional target detection algorithms such as R-CNN [38], SPP-NET [39], and Fast R-CNN [40] possess good recognition rates, but they are poor in real-time because candidate set box extraction and object classification are separate. The objective of semantic segmentation is to assign semantic categories to pixels in an image, such as with SegNet [17], U-Net [41], and FCN [42]. However, the computational complexity is significantly higher due to the use of a fully convolutional structure, which is not suitable for SLAM systems with real-time requirements. If the detection box obtained from object detection is to be used as the a priori information for the SLAM system, it may be necessary to further differentiate between foreground and background by utilizing depth maps and inter-frame information. In contrast, YOLOv8 provides a new SOTA model that efficiently combines target detection and segmentation tasks to achieve accurate segmentation and real-time detection of targets in images. The model extends conventional detection frameworks by incorporating a dedicated segmentation head, enabling simultaneous output of bounding boxes and pixel-level instance masks. This dual-task implementation achieves real-time image analysis while maintaining high segmentation accuracy, ensuring the precise foreground–background separation critical for applications requiring detailed object shape representation. The architecture’s efficiency in balancing computational speed with mask precision makes it particularly suitable for scenarios demanding both real-time performance and accurate spatial delineation of targets. It can be trained on the MS COCO dataset [43]. To achieve real-time performance, the model undergoes TensorRT-based optimization. In this paper, we use the YOLOv8 pixel-level instance segmentation network for semantic information extraction as a priori information for rejecting dynamic objects and constructing semantic point cloud maps. To mitigate the leakage detection of edge pixels, the output mask is appropriately expanded by morphological operations to fully select possible dynamic elements for accurate localization and navigation in dynamic scenes.

### 3.3. Epipolar Constraints

Existing learning-based methods lack robust mechanisms to identify motion in temporarily stationary but inherently dynamic scene elements. Therefore, we use a combination of epipolar geometry constraints and semantic a priori information for the coarse rejection of dynamic features. Non-static keypoints deviate from epipolar constraints due to motion-induced spatial incoherence, as their trajectories fail to align with theoretical epipolar lines following displacement. Therefore, it is possible to determine whether a feature point is a dynamic feature point based on the distance from the feature point to its corresponding pole line. The specific steps of the process are as follows: First, the set of feature matching points for neighboring images is computed using the pyramidal iterative Lucas–Kanade optical flow algorithm [44]. Matching point pairs near image boundaries, exhibiting significant disparities, or situated in regions prone to motion are discarded [19]. Then, the basis matrix is calculated based on the seven-point method of RANSAC. Finally, the basic matrix is then utilized to compute the corresponding polyline for each matching point. Subsequently, the distance between each matching point and its associated polyline is computed and compared against a predefined threshold value. Points are classified as static if their values remain below the threshold; otherwise, they are categorized as dynamic.

**Figure 2:**
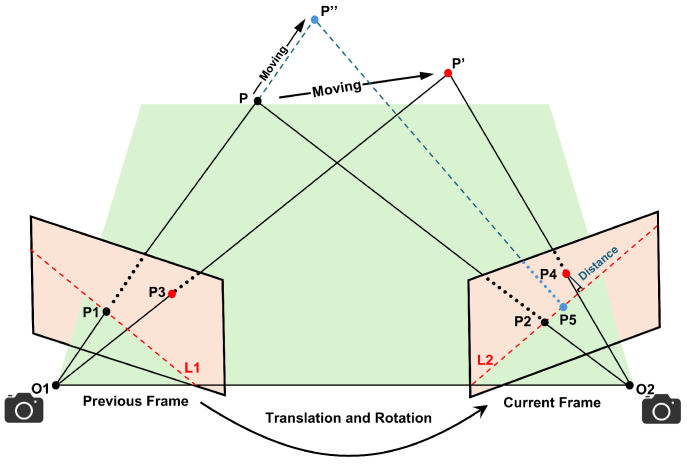
Epipolar geometry constraints. The blue dashed line indicates the situation where the dynamic point is degraded when it moves along the epipolar direction.

Figure 2 represents the epipolar constraints between previous and current frames. According to the pinhole camera model, the camera is rotated and translated to observe spatial points *P*. $O_{1}$ and $O_{2}$ correspond to the camera’s optical centers. $P_{1}$ and $P_{2}$ denote the feature correspondences for the *P* in the prior and current frames, respectively. The red dashed lines $L_{1}$ and $L_{2}$ represent the epipolar lines, while $P^{\prime }$, $P^{\prime \prime }$ indicate the reprojected positions of the displaced map point. Denote $P_{1}$ and $P_{2}$ as follows:

(1) $$P_{1}=\left[\begin{array}{c} x_{1}\\ y_{1} \end{array}\right],\;P_{2}=\left[\begin{array}{c} x_{2}\\ y_{2} \end{array}\right],$$

where *x* and *y* are pixel coordinate values and the corresponding homogeneous coordinate forms for $P_{1}$ and $P_{2}$ should be

(2) $$P_{1}=\left[\begin{array}{c} x_{1}\\ y_{1}\\ 1 \end{array}\right],\;P_{2}=\left[\begin{array}{c} x_{2}\\ y_{2}\\ 1 \end{array}\right].$$

Then, the epipolar line $L_{2}$ in the current frame is subsequently derived from the fundamental matrix *F* by the following equation:

(3) $$L_{2}=FP_{1}=F\left[\begin{array}{c} x_{1}\\ y_{1}\\ 1 \end{array}\right]=\left[\begin{array}{c} X\\ Y\\ Z \end{array}\right],$$

where *X*, *Y*, and *Z* denote the line vectors. As outlined in [25], the epipolar constraint may be expressed in the following manner:

(4) $$P_{2}^{T}FP_{1}=P_{2}^{T}L_{2}=0.$$

The distance *D* between the feature point $P_{2}$ and its associated epipolar line is subsequently defined as follows:

(5) $$D=\frac{\left|P_{2}^{T}FP_{1}\right|}{\sqrt{{∥X∥}^{2}+{∥Y∥}^{2}}}.$$

From the Formula (5), under ideal conditions, the offset distance of the static point *D* should be zero. In real-world scenarios, environmental and sensor noise induce minor displacements, resulting in non-zero deviations that remain bounded within a tolerance threshold. As shown in Figure 2, the point *P* moves to the position of $P^{\prime }$, and the offset distance corresponding to $P_{4}$ will be larger than the threshold and will be considered as a dynamic point rejected. However, dynamic points moving collinearly with the epipolar line create a degenerate case: $P_{5}$ aligns with $L_{2}$ due to the displacement *D* satisfying the static threshold, leading to misclassification as a static feature. So it is necessary to introduce a depth consistency constraint rejection strategy for dynamic rejection.

### 3.4. Depth Consistency Constraint Rejection Strategy

**Figure 3:**
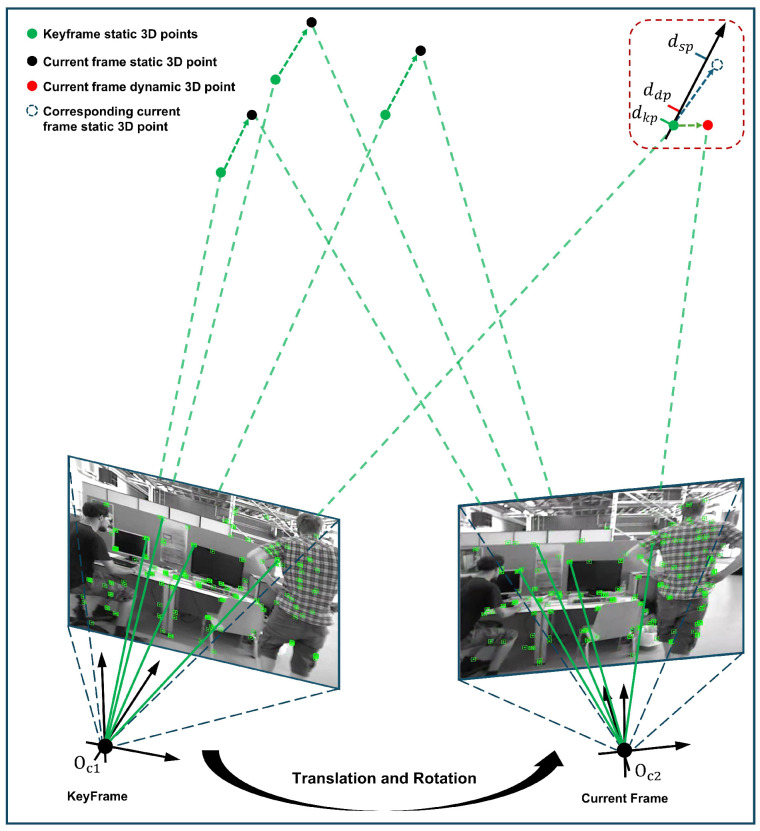
Depth consistency rejection strategy. The red dashed box indicates that the depth difference of the dynamic points in the current frame has changed significantly after being projected onto the keyframe coordinate system. The dynamic points can be removed using a depth consistency rejection strategy.

Due to the complexity of the dynamic environment, there are still limitations in the coarse dynamic feature point rejection methods based on the fusion of semantic and geometric information. Dynamic feature points in the segmentation edge region, points moving along the pole line, and segmentation misdetection points may not be completely rejected. To address these limitations, we introduce a refined rejection strategy that mitigates degenerate cases and enhances both dynamic detection robustness and pose estimation accuracy. The core idea of fine rejection is to reject feature points with large differences by analyzing the consistency between the feature point depth of the current frame and the depth of the corresponding keyframe. For the incoming image frames, after coarse rejection, the remaining feature points are projected to the corresponding keyframes to compute the depth values, and the depth difference between them and the depth map of the keyframes is computed. This constraint is applied by evaluating depth consistency: Feature points with values closer to zero are classified as static, while those exceeding a predefined threshold are identified as dynamic and subsequently discarded. The depth consistency constraint rejection strategy is shown in Figure 3. Green dots denote static 3D points in the keyframe. Black dots indicate the keyframe’s static points projected onto the current frame, while red dots mark dynamic points after projection. Blue dashed dots illustrate the expected depth position of the current frame when projected back to the keyframe. The red dashed box highlights dynamic points exhibiting significant depth deviations, which are filtered out via the depth consistency constraint.

The specific steps are as follows: Given the pixel coordinates of the feature points of the current frame $p={\left[u,v\right]}^{T}$, homogeneous coordinates $p={\left[u,v,1\right]}^{T}$ are constructed. The homogeneous pixel coordinates are mapped to the normalized plane under the normalized camera coordinate system by an inverse projection transformation, where *K* is the camera’s internal reference matrix, which is mathematically represented by the following equation:

(6) $$K=\left[\begin{array}{ccc} f_{x} & 0 & c_{x}\\ 0 & f_{y} & c_{y}\\ 0 & 0 & 1 \end{array}\right].$$

In the internal reference matrix, $f_{x}$ and $f_{y}$ denote the focal lengths along the x-axis and y-axis of the camera, respectively, while $c_{x}$ and $c_{y}$ represent the principal point coordinates marking the intersection of the optical axis with the image plane. The inverse projection transformation is performed by the following equation:

(7) $$\left[\begin{array}{c} x^{\prime }\\ y^{\prime }\\ 1 \end{array}\right]=K^{-1}p_{h}=\left[\begin{array}{ccc} \frac{1}{f_{x}} & 0 & -\frac{c_{x}}{f_{x}}\\ 0 & \frac{1}{f_{y}} & -\frac{c_{y}}{f_{y}}\\ 0 & 0 & 1 \end{array}\right]\cdot \left[\begin{array}{c} u\\ v\\ 1 \end{array}\right]=\left[\begin{array}{c} \frac{u-c_{x}}{f_{x}}\\ \frac{v-c_{y}}{f_{y}}\\ 1 \end{array}\right].$$

The depth observation value $d_{i}\left(p\right)$ is obtained according to the depth map of the current frame, and the normalized plane coordinates calculated by the formula (8) are combined to restore to 3D points $P_{c}$; the formula is as follows:

(8) $$P_{c}=d_{i}\left(p\right)\cdot \left[\begin{array}{c} x^{\prime }\\ y^{\prime }\\ 1 \end{array}\right]=\left[\begin{array}{c} d_{i}\left(p\right)\cdot \frac{u-c_{x}}{f_{x}}\\ d_{i}\left(p\right)\cdot \frac{v-c_{y}}{f_{y}}\\ d_{i}\left(p\right) \end{array}\right].$$

Then, combining the pose of the current frame $T_{i}$ and the pose of the key frame $T_{k}$, the normalized coordinates are mapped to the key frame coordinate system by rigid transformation to obtain 3D points $X_{k}$; the formula is as follows:

(9) $$X_{k}=\left[\begin{array}{c} x_{k}\\ y_{k}\\ z_{k} \end{array}\right]=T_{k}^{-1}T_{i}d_{i}\left(p\right)K^{-1}p_{h}.$$

In order to quantify the depth consistency between ordinary frames and keyframes, 3D point $X_{k}$ needs to be projected to the image plane of keyframes. Then, the corresponding pixel coordinates are obtained by the projection function; the formula is as follows:

(10) $$\pi \left(X_{k}\right)=\left[\begin{array}{c} \frac{x_{k}}{z_{k}}\\ \frac{y_{k}}{z_{k}} \end{array}\right].$$

Then, the depth observation $d_{k}\left(\pi \left(X_{k}\right)\right)$ at the position is extracted in the depth map $d_{k}$ of the keyframe. Ideally, if the depth observation is free of noise and the pose estimation is accurate, the true depth $z_{k}$ of $X_{k}$ should be strictly consistent with the keyframe depth $d_{k}\left(\pi \left(X_{k}\right)\right)$. However, there is noise in the environment, so an empirical value η is set for the reject strategy. The normalized depth residual ε is defined to perform a depth consistency test. The formula is as follows:

(11) $$\varepsilon =\frac{\left(z_{k}-d_{k}\left(\pi \left(X_{k}\right)\right)\right)}{d_{k}\left(\pi \left(X_{k}\right)\right)}.$$

Feature points are classified as dynamic and discarded if the computed |ε| surpasses η.

Keyframes are actively selected by the SLAM system and are usually triggered to be created when there is a significant camera motion or a large change in scene content. Keyframes are retained for long periods and store structured scene information for back-end optimization and loop closure, whereas normal frames only transiently process raw image data to support front-end tracking, and they are typically discarded afterward. Considering that the comparison between consecutive frames may have the problem that the motion is not obvious, the depth information between different frames may have the accumulation of noise. Additionally, the keyframes are high-quality and low-drift frames screened by the system. So we use keyframes to perform the depth consistency constraint test.

### 3.5. Static Point Weighted Optimization

In the back end of the visual SLAM system, this paper proposes an adaptive weighted optimization method based on depth consistency constraints. Through assigning dynamic weights to different qualities of static points, the interference of observation noise on camera pose optimization is effectively suppressed. The core calculation process is as follows. For the pixels $p\in R^{2}$ in the ordinary frame, the depth observation value $d_{i}\left(p\right)$ and the camera intrinsic matrix *K* project these 2D features into 3D normalized camera coordinates. Homogeneous pixel coordinates $p_{h}={\left[u,v,1\right]}^{T}$ are constructed by homogeneous coordinate expansion, and normalized plane coordinates are obtained by inverse projection transformation $K^{-1}p_{h}$. Then, combining the pose of the current frame $T_{i}$ with the keyframe pose $T_{k}$, the normalized coordinates are mapped to the keyframe coordinate system by rigid transformation $T_{k}^{-1}T_{i}$, and the 3D point $X_{k}$ is finally obtained. This process achieves geometrically consistent alignment across frames and lays the foundation for static weight calculation.

The keyframe depth observations $d_{k}\left(\pi \left(X_{k}\right)\right)$ obtained from the previous section should ideally agree strictly with the true depth $z_{k}$ of $X_{k}$. Therefore, the normalized depth residual ε is introduced, and the physical meaning is a proportional measure of the relative depth deviation. Compared to the absolute error, the normalized form can deal with the observation uncertainty at different distances in a more balanced way, avoiding the over-penalization of distant points due to the large absolute error.

Based on the above residuals, the adaptive weight function is further designed to dynamically adjust the confidence level of each observation. An exponential weighting model $\omega \left(p\right)=\exp\left(-\lambda \varepsilon^{2}\right)$ is used, in which hyperparameters, λ control the decay rate of weights with depth deviation. The complete weighting formula is as follows:

(12) $$\omega \left(p\right)=\exp\left(-\lambda \cdot {\left(\frac{z_{k}-d_{k}\left(\pi \left(X_{k}\right)\right)}{d_{k}\left(\pi \left(X_{k}\right)\right)}\right)}^{2}\right).$$

When the depth consistency error ε approaches zero, the weight ω(p) approaches the maximum value of 1, indicating that the observation is highly reliable. With the increase in ε, the weight decays rapidly in a Gaussian pattern, effectively suppressing the interference of abnormal observations caused by dynamic objects, depth estimation errors, or pose drift in the optimization process. This weight function is directly applied to the information matrix and adjusts the contribution of each residual term to the overall optimization through weighting. This relationship is mathematically formulated as follows:

(13) $$\Omega_{i}=\omega \left(p\right)\cdot I_{2\times 2}=\exp\left(-\lambda \cdot {\left(\frac{z_{k}-d_{k}\left(\pi \left(X_{k}\right)\right)}{d_{k}\left(\pi \left(X_{k}\right)\right)}\right)}^{2}\right)I_{2\times 2}.$$

Observations with higher depth consistency are given greater weights to strengthen their constraints in parameter optimization, while those with lower consistency have their influence reduced through weight decay. The method indirectly suppresses the error propagation of dynamic points or low-quality static points through geometric transformations, depth consistency verification, and statistical weighting mechanisms. It significantly enhances the resilience of the system to sensor noise and dynamic disturbances, while avoiding the overfitting or underconstraint problems caused by fixed weights or threshold settings in traditional methods.

### 3.6. Dense Mapping

**Figure 4:**
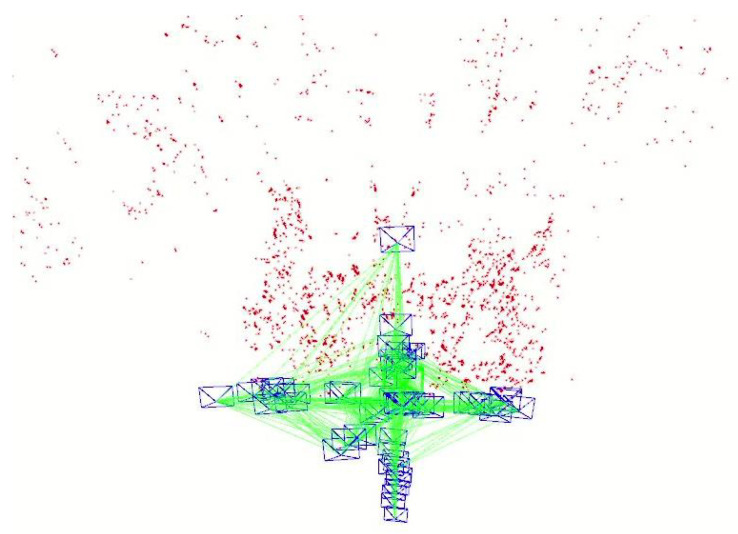
Schematic of sparse feature points.

The core output of original ORB-SLAM2 [7] is a sparse feature point map (for pose estimation and loop detection). Figure 4 illustrates the sparse feature point map of the fr3_walking_xyz sequence of the TUM dataset. Therefore, SGDO-SLAM adds a dense point cloud mapping thread that removes the influence of dynamic objects. This thread generates dense point clouds with accurate depth information from RGB images and depth maps.

**Figure 5:**
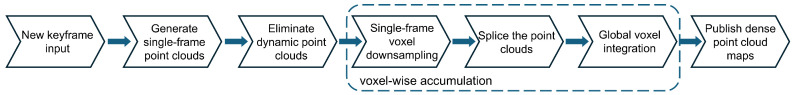
Schematic of the dense mapping method. The dashed box indicates the process of voxel-wise accumulation.

Figure 5 shows the process of dense mapping. Firstly, single-frame point cloud generation is performed according to the input new keyframe. Then, the dynamic region point cloud is eliminated according to the semantic prior information. To reduce single-frame point cloud redundancy, single-frame voxel downsampling is performed. The global point cloud map maintenance of SGDO-SLAM adopts the key frame trigger update mechanism. When the cumulative number of frames reaches a threshold, statistical outlier removal is combined with voxel grid sampling to improve map accuracy and maintain density balance. Finally, 3D point cloud data conforming to the standard coordinate system specification are released through ROS to realize real-time map updates and visualization.

The dense mapping process combines the information of the instance segmentation thread, removes the mapping points associated with the potential moving objects in the point cloud image, and only preserves the mapping points of the objects with static attributes, which ensures the accurate construction of the dense point cloud. It can save the object information segmented in the segmentation thread to establish a point cloud map with semantic information. The dense point cloud map provides richer geometric information and details of the environment, which can assist the robot in completing some more complex tasks.

## 4. Experimental Results

To assess the system’s efficacy, experiments are conducted on the TUM RGB-D dataset [45] and the Bonn dataset [46]. In our experiments, localization accuracy is quantified using the absolute trajectory error (ATE) and relative pose error (RPE). The root mean square error (RMSE) and standard deviation (S.D.) are commonly used to characterize the accuracy and stability of the system, respectively. The RPE consists of the relative translation drror (RTE) and relative rotation error (RRE). First, SGDO-SLAM is compared with the classical algorithm ORB-SLAM2 and other state-of-the-art dynamic SLAM systems in two open-source datasets. Second, ablation studies validate the fusion framework’s superiority relative to standalone methods. Then, the operational efficiency of the system is evaluated. All experiments are conducted on a laptop with a Ubuntu 20.04 operating system, a NVIDIA GeForce RTX 2060 GPU, an AMD Ryzen 7 4800H CPU, and 16GB of RAM.

### 4.1. Performance Evaluation on TUM RGB-D Dataset

In this section, to assess the SGDO-SLAM system’s accuracy in dynamic environments, experiments are performed on the TUM RGB-D dataset, including four high-dynamic sequences (fr3_walking_xyz, fr3_walking_rpy, fr3_walking_half, and fr3_walking_static) and one low-dynamic sequence (fr3_sitting_static). Walking and sitting denote the scenes of a person walking in an image sequence and a person sitting in an image sequence, respectively. The labels xyz, static, rpy, and half correspond to distinct camera movement patterns.

#### 4.1.1. Comparison of Baseline Algorithm

To assess the advancements of SGDO-SLAM over the baseline, we perform an experimental comparison with ORB-SLAM2. Table 1, Table 2 and Table 3 present a quantitative comparison of trajectory estimation accuracy between SGDO-SLAM and ORB-SLAM2 across five TUM sequences, evaluating both the absolute trajectory error (ATE) and relative pose error components (translational/rotational drift).

**Figure 6:**
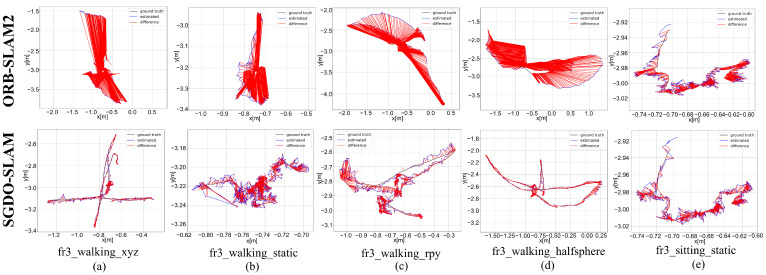
Comparative ATE metrics for SGDO-SLAM and ORB-SLAM2 across five sequences. (**a**) fr3_walking_xyz. (**b**) fr3_walking_static. (**c**) fr3_walking_rpy. (**d**) fr3_walking_halfsphere. (**e**) fr3_sitting_static.

**Figure 7:**
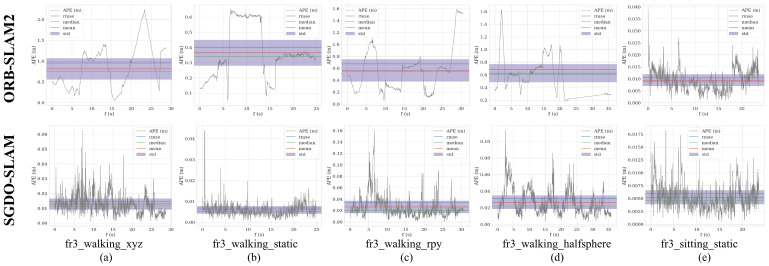
Comparative ATE metrics for SGDO-SLAM and ORB-SLAM2 across five sequences. (**a**) fr3_walking_xyz. (**b**) fr3_walking_static. (**c**) fr3_walking_rpy. (**d**) fr3_walking_halfsphere. (**e**) fr3_sitting_static.

The results in Table 1, Table 2 and Table 3 show that the precision of SGDO-SLAM on different sequences is improved to different degrees compared to ORB-SLAM2. In addition, the four metrics (RMSE, mean, median, and S.D.) in ATE are increased by 98.46%, 98.44%, 98.62%, and 98.64% at most. In low dynamic sequences, the lift is small compared to dynamic sequences because of spatial range constraints and the limited range of moving objects. In the ATE four metrics (RMSE, mean, median and S.D.), it increased by 44.34%, 44.08%, 48.31%, and 45.83%. The ATE schematics of ORB-SLAM2 and SGDO-SLAM on five sequences are shown in Figure 6 and Figure 7. In Figure 6, the gray trajectory denotes the ground truth, the blue curve illustrates the estimated pose, and the red region highlights the positional deviation between them. While ORB-SLAM2 exhibits significant drift in highly dynamic environments, our approach maintains trajectories that align closely with the ground truth. Figure 7 shows the change curve of ATE. SGDO-SLAM achieves a substantial reduction in trajectory estimation errors and demonstrates significantly lower variability compared to ORB-SLAM2. This experiment demonstrates that the introduction of dynamic reject strategy and static point back-end optimization improvement effectively elevates the precision of the framework.

#### 4.1.2. Comparison of State-of-the-Art Algorithms

To further evaluate the effectiveness of the algorithm, we test SGDO-SLAM with ORB-SLAM3 [9] and state-of-the-art SLAM systems, such as SG-SLAM [11], COEB-SLAM [47], and YOLO-SLAM [20], on five sequences of the TUM dataset, and the results of the experiments are shown in Table 4, with the optimal data are bolded in the table. In dynamic environments, ORB-SLAM3 exhibits persistent localization inaccuracies, whereas SGDO-SLAM suppresses the impact of moving objects and enhances positional reliability. In the fr3_walking_rpy sequence, most of the algorithms do not work very well on this sequence due to the blurring of the image resulting from rapid rotational motion in the camera. DynaSLAM, because of the image complementation with multiview geometry, has a higher accuracy under the influence of large areas as well as repetitive dynamic objects. The SGDO-SLAM algorithm’s accuracy for this sequence is second only to that of DynaSLAM. The static weighting optimization enhances localization precision by prioritizing contributions from reliable static features during pose estimation. Comparing the SG-SLAM algorithm with the data in Table 4, our algorithm has improved accuracy and robustness in all sequences. This is because the depth consistency constraints can better reject the influence of dynamic points that move along the poles, are on the edge, or have segmentation errors. Finally, SGDO-SLAM has the best average accuracy among the five sequences and possesses the best accuracy and robustness among the four sequences, further demonstrating the algorithm’s dynamics-awareness and effectiveness.

**Figure 8:**
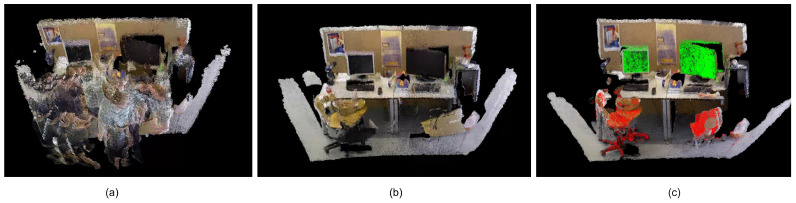
TUM dataset dense point cloud construction results. (**a**) fr3_walking_xyz’s dense map without dynamic objects removed. (**b**) fr3_walking_xyz’s dense map with dynamic objects removed. (**c**) fr3_walking_xyz’s semantic point cloud map.

Figure 8 illustrates a comparative analysis of 3D reconstruction fidelity for the TUM dataset’s fr3_walking_xyz dynamic sequence. Figure 8a shows the map building results without removing the dynamic objects, where the point clouds on the dynamic objects are also added to the map building, resulting in a lot of human drag shadows in the map. Figure 8b visualizes the high-fidelity 3D reconstruction after mitigating dynamic elements, highlighting the system’s robustness to motion artifacts. Figure 8c shows the map, which highlights the recognized static objects by detecting the information of threads, thereby enhancing the robot’s ability to interpret and navigate complex environments. The monitor and chair are shown with green and red point clouds, respectively.

### 4.2. Performance Evaluation on Bonn RGB-D Dataset

Introduced by Bonn University in 2019, the Bonn RGB-D dataset [46] comprises 24 dynamic sequences for benchmarking RGB-D SLAM systems in motion-rich environments. To evaluate our method’s efficacy and adaptability, nine representative sequences are selected for experimentation. The “Crowd” sequence depicts a dynamic scene with three individuals navigating a confined space. The “Moving no box” sequence demonstrates object transfer from a lower to an elevated surface (floor to table). The “Person tracking” sequence evaluates camera tracking capabilities under slow pedestrian motion. The “synchronous” sequence shows several people repeatedly jumping in the same direction. These nine sequences have more complex dynamics than the TUM dataset and are more challenging for SLAM systems.

Table 5 summarizes the experimental outcomes, including quantitative metrics for accuracy across all sequences. We compare ORB-SLAM2 and the current advanced algorithms YOLO-SLAM [20], SG-SLAM [11,48], and DN-SLAM [49]. A “-” in the table means that the algorithm does not provide data. From the experimental data, SGDO-SLAM does not perform as well on “Crowd1” sequence as [48]. Because Huai et al. [48] uses the dynamic semantic compensation mechanism, probabilistic feature point filtering targeted to solve the dynamic fuzzy problem, the performance is better on sequences with a lot of fuzzy interference. However, from an overall perspective, SGDO-SLAM’s performance remains superior to their system. In the “Crowd3” sequence, the performance of SGDO-SLAM is not as good as that of DN-SLAM, because the unpredictable trajectories of persons in “Crowd3” lead to widespread dispersion of dynamic features, which occupy a significant portion of the image frame, while DN-SLAM utilizes the dynamic density of the dynamic points to eliminate the dynamic effects. DN-SLAM [49] employs a dynamic density-adaptive feature point optimization method and NeRF background restoration to maintain a sufficient density of static features to sustain robust pose estimation. In the “Synchronous2” sequence, the localization accuracy of SGDO-SLAM is slightly inferior to that of YOLO-SLAM, which effectively reduces the pose drift due to the synchronous motion through the dynamic point rejection assisted by the optical flow method. In contrast, our algorithm’s depth consistency constraints can induce misclassification during camera-synchronized dynamic object motion, degrading pose estimation accuracy. Compared to SG-SLAM, in the two “synchronization” sequences, our algorithm solves the degeneracy problem when the dynamic features move in a similar direction to the pole lines, and it improves the localization accuracy by 97.12% and 73.81%, respectively.

**Figure 9:**
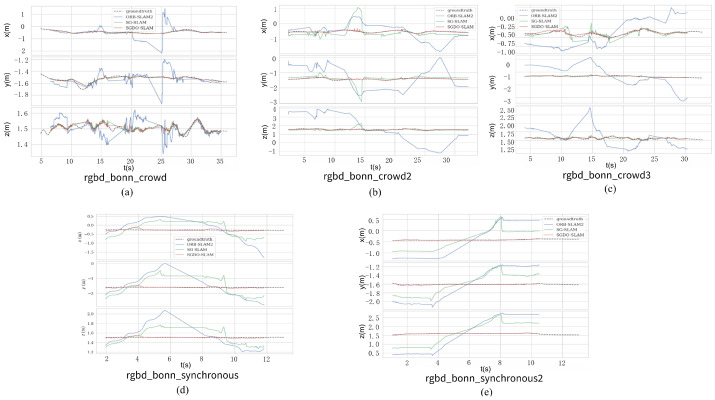
Results of xyz-axis trajectories for partial sequences of the Bonn dataset. (**a**) rgbd_bonn_crowd. (**b**) rgbd_bonn_crowd2. (**c**) rgbd_bonn_crowd3. (**d**) rgbd_bonn_synchronous1. (**e**) rgbd_bonn_synchronous2.

Figure 9 illustrates trajectory plots for a single axis for the partial sequence in the Bonn dataset. In the Figure 9, red, green, and blue denote the trajectories of SGDO-SLAM, SG-SLAM, and ORB-SLAM2, respectively, with the ground truth represented by a black dashed line. From the figure, it can be seen that ORB-SLAM2 drifts badly in all sequences, while SGDO-SLAM has a very good performance in all sequences and all converge to the ground truth. Then, in Figure 9d,e it is shown that when SG-SLAM encounters an object moving along the pole line, the trajectory of the pose estimation is difficult to converge to the ground truth. SGDO-SLAM solves this problem by using the depth-consistent constraint rejection strategy, and the pose estimation results fit the ground truth. SGDO-SLAM achieves the highest accuracy and a relatively low standard deviation in most sequences due to the static weight optimization method, which indirectly suppresses the propagation of errors from dynamic or low-quality static points. This experiment demonstrates that SGDO-SLAM can achieve high accuracy in highly dynamic scenarios with good generalization performance.

**Figure 10:**
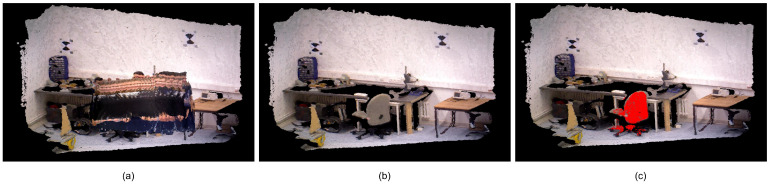
Bonn dataset dense point cloud construction results. (**a**) synchronous2’s dense map without dynamic objects removed. (**b**) synchronous2’s dense map with dynamic objects removed. (**c**) synchronous2’s semantic point cloud map.

Figure 10 demonstrates the comparison of dense point cloud construction results for synchronous2 sequences in the Bonn dataset. Figure 10a shows the map construction results without removing dynamic objects, and there is a large area of dragging shadow interfering with the acquisition of environmental information. Figure 10b illustrates the refined dense point cloud map after dynamic object suppression, resulting in enhanced environmental reconstruction fidelity and superior map integrity. The point cloud of the chair is labeled in Figure 10c, providing richer map information.

### 4.3. Ablation Experiment

Ablation studies are performed to evaluate the efficacy of the fusion framework. First, SGDO-SLAM (G) denotes the dynamic feature point rejection using only epipolar geometric constraints. SGDO-SLAM (O) performs only static point weight optimization. SGDO-SLAM (S+G) is an algorithm using only segmentation threads and epipolar geometric constraints, and SGDO-SLAM (S+G+D) is a combination of segmentation thread and epipolar geometric constraints algorithms and a depth consistent rejection strategy. SGDO-SLAM (S+G+O) uses coarse dynamic feature rejection and static point weight optimization. Finally, there is SGDO-SLAM (S+G+D+O), an algorithm that combines instance segmentation, epipolar geometric constraints, depth consistent rejection, and a static point weight optimization strategy. We still conduct experiments on five sequences of the TUM dataset [45] using the two metrics of ATE (RMSE and S.D.) for quantitative evaluation. From the experimental data in Table 6, it can be seen that a single geometric constraint is difficult to deal with complex motion disturbances effectively, while the introduction of the instance segmentation module can effectively differentiate between dynamic and static regions and reduce mismatches. A single static weight optimization without the aid of a reject strategy will include a portion of mis-estimation of dynamic features leading to a decrease in system accuracy. Then, the addition of a depth consistent rejection strategy increases the robustness of rejecting dynamic features. Finally, the addition of back-end static point weight optimization further suppresses the noise interference and improves the anti-interference capability of the system. In Table 6, it can be seen that SGDO-SLAM (S+G+D+O) is the most accurate in the measured sequence experiments, proving the effectiveness of the fusion algorithm.

**Figure 11:**
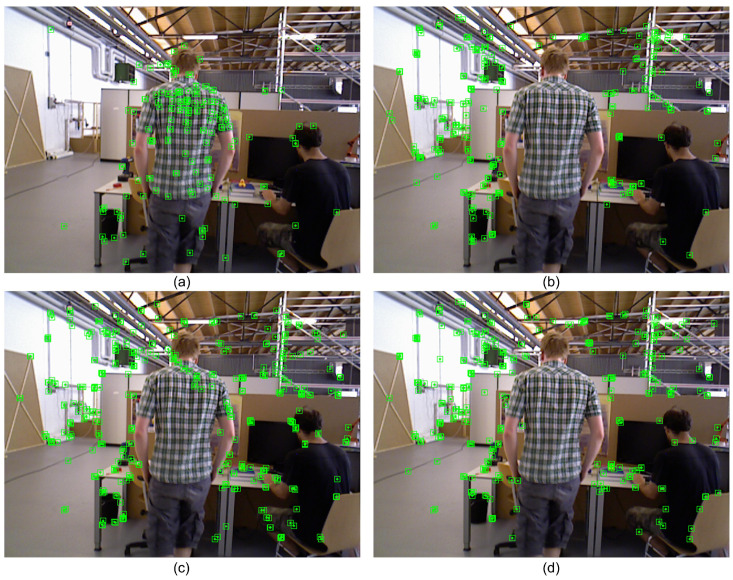
Dynamic feature rejection effect demonstration. (**a**) ORB-SLAM2. (**b**) SGDO-SLAM (S). (**c**) SGDO-SLAM (S+G). (**d**) SGDO-SLAM (S+G+D).

Figure 11a shows the result of ORB feature point extraction. The feature points of the dynamic part are basically not eliminated, and most of them are distributed on dynamic objects. Figure 11b shows the result of applying only instance segmentation. Compared to the violent rejection of object detection, the rejection of instance segmentation utilizing masks is more refined, but there are cases where static points at the edges are rejected. Figure 11c shows the dynamic feature rejection results after combining the segmentation results with the geometric constraints. With the addition of the geometric constraint method, which no longer relies on the mask for violent rejection, more feature points at the edges are retained. However, there will be a small portion of dynamic points not rejected cleanly due to the threshold setting or the degradation of the object’s motion along the polar direction. Figure 11d shows the experimental results with the introduction of a depth consistency constraint rejection strategy based on the combination of semantics and geometry. While maintaining the fine extraction of edge points, the dynamic feature points of false detection and missed detection are eliminated, and the elimination effect is more accurate and fine than that of the previous algorithm. The quantitative and qualitative results in Figure 11 and Table 6 validate the efficacy of the SGDO-SLAM fusion framework.

### 4.4. Time Analysis

To evaluate the system’s real-time capability and to ensure that the system can perform its tasks properly in the environment, we tested the average time overhead of processing each frame and compared it to other advanced systems. Table 7 summarizes the per-frame computational latency and hardware configuration. Table 7 demonstrates that SGDO-SLAM incurs only a marginal per-frame latency increase (<10 ms) over ORB-SLAM2, achieving substantial accuracy gains without sacrificing real-time performance. While YOLO-SLAM and Dyna-SLAM sacrifice speed for precision segmentation, SGDO-SLAM attains modest accuracy enhancements with markedly lower computational overhead. SGDO-SLAM demonstrates robust performance in experimental evaluations, maintaining real-time operation suitable for robotic applications.

## 5. Conclusions

In this paper, a real-time semantic visual SLAM (SGDO-SLAM) system is proposed. A coarse-to-fine dynamic rejection method is proposed to solve the degradation problem of non-potentially moving objects moving along the polar direction and enhance the system’s capability to detect changing surroundings. The method first combines semantic information and geometric information for coarse rejection, then performs fine rejection by depth consistency constraints and calculates static quality weights. Then, a pose optimization method based on static quality weights is proposed to utilize high-quality static features for back-end optimization, which boosts pose estimation reliability and constructs more precise environmental representations. The experimental evaluations demonstrate that SGDO-SLAM achieves superior localization precision and operational stability in highly dynamic scenes compared to ORB-SLAM2 and state-of-the-art dynamic SLAM frameworks, while maintaining real-time performance, critical for the reliable execution of autonomous robotic missions. In the future, we will introduce additional sensors to better handle complex environmental disturbances. There is also a need to create maps with smaller footprints and more differentiated information to increase the intelligent perception of the robot.

## Figures and Tables

**Table 1:** Absolute trajectory error (ATE) comparison between the proposed method and baseline on the TUM dataset. (Unit: m).

Sequences	ORB-SLAM2				SGDO-SLAM (Ours)				Improvements			
	RMSE	Mean	Median	S.D.	RMSE	Mean	Median	S.D.	RMSE (%)	Mean (%)	Median (%)	S.D. (%)
fr3_walking_xyz	0.9574	0.8148	0.7589	0.5027	0.0147	0.0127	0.0105	0.0068	98.46	98.44	98.62	98.64
fr3_walking_static	0.4021	0.3648	0.3399	0.1689	0.0068	0.0059	0.0052	0.0034	98.31	98.38	98.47	97.99
fr3_walking_rpy	0.6726	0.5642	0.5506	0.3661	0.0339	0.0263	0.0201	0.0213	94.96	95.34	96.35	94.18
fr3_walking_half	0.6871	0.6271	0.6021	0.2807	0.0258	0.0223	0.0182	0.0122	96.25	96.44	96.98	95.65
fr3_sitting_static	0.0106	0.0093	0.0089	0.0048	0.0059	0.0052	0.0046	0.0026	44.34	44.08	48.31	45.83

**Table 2:** Translational drift (RPE) evaluation comparison between the proposed method and baseline on the TUM dataset. (Unit: m/s).

Sequences	ORB-SLAM2				SGDO-SLAM (Ours)				Improvements			
	RMSE	Mean	Median	S.D.	RMSE	Mean	Median	S.D.	RMSE (%)	Mean (%)	Median (%)	S.D. (%)
fr3_walking_xyz	0.5363	0.4245	0.5044	0.3276	0.0175	0.0152	0.0137	0.0085	96.73	96.41	97.28	97.40
fr3_walking_static	0.3063	0.1546	0.0265	0.2643	0.0064	0.0060	0.0062	0.0019	97.91	96.11	76.60	99.28
fr3_walking_rpy	0.4902	0.3568	0.2622	0.3361	0.0473	0.0380	0.0297	0.0281	90.35	89.34	88.67	91.63
fr3_walking_half	0.6580	0.4676	0.2569	0.4628	0.0374	0.0328	0.0265	0.0179	94.31	92.98	89.68	96.13
fr3_sitting_static	0.0148	0.0111	0.0087	0.0098	0.0084	0.0072	0.0066	0.0044	43.24	35.13	24.13	55.10

**Table 3:** Rotational drift (RPE) evaluation comparison between the proposed method and baseline on the TUM dataset. (Unit: °/s).

Sequences	ORB-SLAM2				SGDO-SLAM (Ours)				Improvements			
	RMSE	Mean	Median	S.D.	RMSE	Mean	Median	S.D.	RMSE (%)	Mean (%)	Median (%)	S.D. (%)
fr3_walking_xyz	10.5042	8.2556	9.4303	6.4947	0.4694	0.4377	0.4221	0.1696	95.53	94.69	95.52	97.38
fr3_walking_static	5.5499	2.8426	0.5174	4.7666	0.2524	0.2324	0.2150	0.0985	95.45	91.82	58.44	97.93
fr3_walking_rpy	9.2766	6.8413	4.1514	6.2651	0.8542	0.7117	0.5411	0.4723	90.79	89.59	86.96	92.46
fr3_walking_half	14.7136	10.5943	5.5201	10.2103	0.8603	0.7729	0.6321	0.3777	94.15	92.70	88.54	96.30
fr3_sitting_static	0.3969	0.3350	0.3343	0.2128	0.2482	0.2649	0.2469	0.1458	27.46	25.91	20.75	31.48

**Table 4:** Absolute trajectory error (ATE) comparison between the proposed method and advanced methods on the TUM dataset. (Unit: m).

Seq.	YOLO-SLAM		DS-SLAM		SG-SLAM		COEB-SLAM		RDS-SLAM		DynaSLAM		ORB-SLAM3		SGDO-SLAM (Ours)	
	RMSE	S.D.	RMSE	S.D.	RMSE	S.D.	RMSE	S.D.	RMSE	S.D.	RMSE	S.D.	RMSE	S.D.	RMSE	S.D.
fr3_w_xyz	0.0146	0.0070	0.0247	0.0161	0.0184	0.0096	0.0188	0.0092	0.0571	0.0229	0.0156	0.0079	0.7012	0.3018	**0.0131**	**0.0068**
fr3_w_static	0.0073	0.0035	0.0081	0.0067	0.0076	0.0037	0.0073	0.0034	0.0206	0.0120	0.0079	0.0043	0.4218	0.2246	**0.0068**	**0.0034**
fr3_w_rpy	0.2164	0.1001	0.4442	0.2350	0.0386	0.0233	0.0364	0.0227	0.1604	0.0874	**0.0325**	**0.0194**	0.8656	0.4526	0.0339	0.0213
fr3_w_half	0.0283	0.0138	0.0303	0.0283	0.0300	0.0156	0.0315	0.0148	0.0807	0.0454	0.0261	0.0123	0.6280	0.2926	**0.0188**	**0.0092**
fr3_s_static	0.0066	0.0033	0.0065	0.0033	0.0063	0.0030	0.0075	0.0037	0.0084	0.0043	0.0067	0.0028	0.0090	0.0043	**0.0059**	**0.0026**
Mean	0.0546	0.0255	0.1027	0.0578	0.0201	0.0110	0.0203	0.0107	0.0654	0.0344	0.0177	0.0093	0.5251	0.2551	**0.0157**	**0.0086**

**Table 5:** Absolute trajectory error (ATE) comparison between the proposed method and advanced methods on the Bonn dataset. (Unit: m).

Seq.	ORB-SLAM2		YOLO-SLAM		SG-SLAM		DN-SLAM		Huai et al. [48]		SGDO-SLAM (Ours)	
	RMSE	S.D.	RMSE	S.D.	RMSE	S.D.	RMSE	S.D.	RMSE	S.D.	RMSE	S.D.
crowd1	0.8632	0.5918	0.033	-	0.0234	0.0143	0.025	0.016	**0.016**	**0.008**	0.0206	0.0126
crowd2	1.3573	0.6207	0.423	-	0.0584	0.0406	0.028	0.017	0.031	0.018	**0.0278**	**0.0148**
crowd3	1.0772	0.3823	0.069	-	0.0319	0.0219	**0.026**	**0.014**	0.026	0.017	0.0290	0.0157
moving_no_box1	0.1174	0.0710	0.027	-	0.0192	**0.0081**	0.026	0.014	-	-	**0.0014**	0.0093
moving_no_box2	0.1142	0.0598	0.035	-	0.0299	**0.0030**	0.120	0.061	-	-	**0.0246**	0.0095
person_tracking1	0.7959	0.3617	0.157	-	0.0400	0.0139	0.038	0.015	0.038	0.012	**0.0232**	**0.0101**
person_tracking2	1.0679	0.4699	0.037	-	0.0376	0.0154	0.042	0.017	0.034	**0.013**	**0.0301**	0.0140
synchronous1	1.1411	0.5703	0.014	-	0.3229	0.1824	-	-	-	-	**0.0093**	**0.0046**
synchronous2	1.4069	1.3201	**0.007**	-	0.0164	0.0126	-	-	-	-	0.0071	**0.0039**

**Table 6:** Absolute trajectory error (ATE) comparison between different modules on the TUM dataset. (Unit: m).

Seq.	SGDO-SLAM (G)		SGDO-SLAM (O)		SGDO-SLAM (S+G)		SGDO-SLAM (S+G+O)		SGDO-SLAM (S+G+D)		SGDO-SLAM (S+G+D+O)	
	RMSE	S.D.	RMSE	S.D.	RMSE	S.D.	RMSE	S.D.	RMSE	S.D.	RMSE	S.D.
fr3_w_xyz	0.0416	0.0130	0.0346	0.0096	0.0157	0.0079	0.0142	0.0076	0.0148	0.0076	**0.0131**	**0.0068**
fr3_w_static	0.0226	0.0102	0.0165	0.0088	0.0071	0.0036	0.0080	0.0038	0.0081	0.0040	**0.0068**	**0.0034**
fr3_w_rpy	0.1333	0.0675	0.0862	0.0468	0.0412	0.0256	0.0393	0.0248	0.0379	0.0238	**0.0339**	**0.0213**
fr3_w_half	0.0375	0.0241	0.0246	0.0198	0.0231	0.0113	0.0222	0.0096	0.0204	0.0101	**0.0188**	**0.0092**
fr3_s_static	0.0072	0.0036	0.0071	0.0030	0.0062	0.0029	0.0060	0.0027	0.0060	0.0027	**0.0059**	**0.0026**

**Table 7:** Average processing time per frame for different systems.

Systems	Average Processing Time per Frame (ms)	Hardware Platform
ORB-SLAM2	31.21	AMD Ryzen 7 4800H CPU, NVIDIA RTX 2060 GPU
SG-SLAM	40.61	AMD Ryzen 7 4800H CPU, NVIDIA RTX 2060 GPU
COEB-SLAM	50.63	AMD Ryzen 7 4800H CPU, NVIDIA RTX 2060 GPU
YOLO-SLAM	696.09	Intel Core i5-4288U CPU
DynaSLAM	195.00	Intel i7 CPU, P4000 GPU
VINS-Fusion	57.50	i7-9700K CPU, Nvidia RTX 2080, 48GB RAM
SGDO-SLAM(ours)	38.81	AMD Ryzen 7 4800H CPU, NVIDIA RTX 2060 GPU

## Data Availability

The original contributions presented in this study are included in the article. Further inquiries can be directed to the corresponding author.
